# Orthodontic treatment of the transposition of a maxillary canine and a first premolar: a case report

**DOI:** 10.1186/s13256-015-0521-z

**Published:** 2015-03-01

**Authors:** Dinoi Maria Teresa, Mummolo Stefano, Monaco Annalisa, Marchetti Enrico, Campanella Vincenzo, Marzo Giuseppe

**Affiliations:** 1 Department MeSVA, School of Dentistry, University of L’Aquila, L’Aquila, Italy; 2 Department of Clinical Science and Translational Medicine, University of Rome “Tor Vergata”, Rome, Italy

**Keywords:** Canine, First premolar, Orthodontics, Transposition

## Abstract

**Introduction:**

Transposition is an anomaly of tooth position, the most frequent of which involves the canine and the first maxillary premolar. We describe the orthodontic treatment of a unilateral transposition of an upper canine and an upper right first premolar in the permanent dentition.

**Case presentation:**

A 12-year-old Caucasian boy presented with transposition of his upper right canine and upper right first premolar. He had combined surgical-orthodontic treatment to correct the transposition and to obtain a Class I relationship between the molar and canine. This treatment resolved the dental crowding and achieved good functional and aesthetic results.

**Conclusion:**

In transposition, the choice of the most suitable treatment depends on the occlusion, level of dental crowding, aesthetics, position of the radicular apices, and the specific needs of the patient. In this case, orthodontic alignment of the transposed teeth into their physiological position achieved all of our objectives and our patient was satisfied with the aesthetic results obtained.

## Introduction

Transposition is a form of ectopic eruption, defined as the positional interchange of two adjacent teeth within the same quadrant of the dental arch [1,2]. Transposition can be complete or incomplete. In complete transposition, the entire dental structure (root and crown) is in an ectopic position. In incomplete transposition (also called pseudo- or partial transposition), the crowns are ectopic, but the roots are in the correct position [1].

Transpositions mostly involve the upper arch and are unilateral [3-5]. To the best of our knowledge, transposition has never been observed in both dental arches or in the deciduous dentition [1,6]. By analyzing a sample of 201 cases, Peck and Peck [2] identified five types of transposition: upper canine-first premolar; upper canine-lateral incisor; upper canine-first molar; upper lateral incisor-central incisor; and lower lateral incisor-canine [3]. Of all the teeth, the permanent maxillary canines are the most frequently transposed. The most common type of transposition is between the canine and first maxillary premolar, followed by transposition between the canine and the lateral incisor, central incisor, second premolar, and first premolar in the lower arch [1-3,7]. On the transposed side, it is not unusual to find agenesis of the lateral incisors and second premolars, or to find inclusions of the canines and central incisors [5,6,8-10]. Microdontia also very frequently occurs with transpositions.

Transposition between the canine and the first maxillary premolar occurs in 0.135% to 0.510% of the population [4,6,11]. In Japan, the incidence ranges from 0.065% in the general population to 0.660% in orthodontic patients [11-14]. Elsewhere, occurrence ranges from 0.380% in a Turkish population [11,15] to 0.510% in Africa [11,16]. In a study of 2349 children between 2 and 12 years of age, Buenviaje and Rapp found that the prevalence of transposed teeth was 0.080% [11,17].

This type of transposition shows the following characteristics [3]: the deciduous canine is present; the canine is positioned between the first and second premolars; the canine is positioned towards the vestibule; the first premolar is mesiopalatal; and the transposition area shows dental crowding, especially if the deciduous canine is present.

Postulated causes of tooth transposition include inversion of the tooth buds during development [4,8,15,18], alteration of the tooth eruption pattern [4,15,18,19], the presence of deciduous teeth beyond the maximum time limit for the development of the permanent teeth [4,15,18,20], and dental trauma during childhood [4,15,18,21]. However, evidence also exists for genetic factors, including the increased prevalence of transposition in females [1,8], on the left side [1,8], and in patients with hypodontia [1,8] or Down Syndrome [22]. Accordingly, it has been suggested that the etiology of tooth transposition has a genetic basis, within a picture of multifactorial heredity [3,8,23-25].

The treatment of transpositions can be classified as interceptive or definitive, depending on when the transposition is diagnosed, although in some cases these two treatment types can overlap [3,9]. Interceptive treatment is performed on patients between six and eight years of age after orthopantomography of the dental arches and a thorough intraoral examination reveal the presence of tooth transposition at the initial stage. This treatment involves extraction of the retained deciduous teeth, positioning of the permanent lateral incisor in its physiological position, and maintenance of the space for the permanent canine. Interceptive treatment can be adopted before transposition is complete, which normally occurs around 10 years of age. After that time, definitive treatment should be adopted [3,9]. Definitive treatment involves three steps: extraction of one of the transposed teeth, alignment of the teeth in the transposed position, and orthodontic correction and alignment of teeth in the correct position [26,27]. The decision is affected by several factors, such as the patient’s degree of occlusion and dental crowding, aesthetics, the position of the radicular apices, socio-economic factors, and the patient’s motivation.

We present the case of a patient with transposition between the canine and first premolar, demonstrating three of the typical characteristics: he still had the deciduous canine; orthopantomography determined that the canine was positioned between the first and second premolars; and clinical observation showed that the canine was positioned towards the vestibule. We successfully treated the transposition with definitive treatment.

## Case presentation

**Figure 1:**
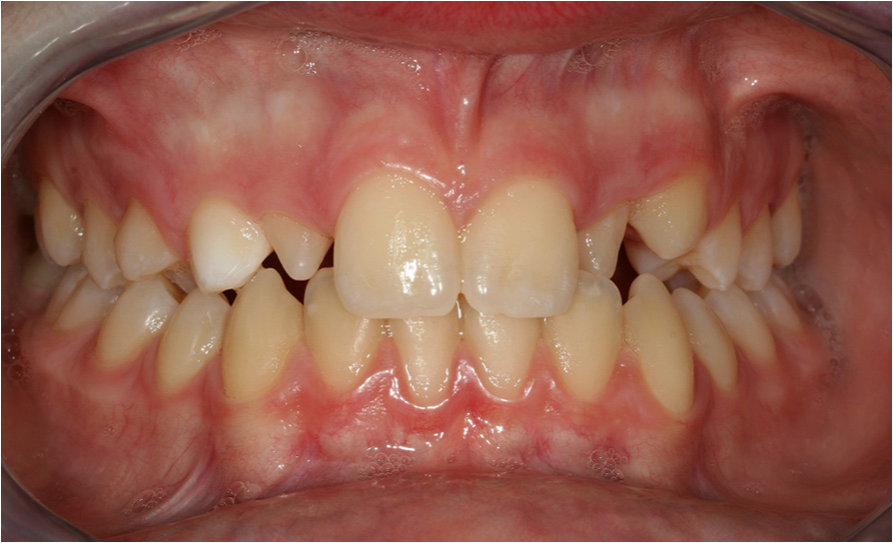
**Pretreatment intraoral frontal radiograph.**

We describe the case of a 12-year-old male Caucasian patient in the permanent dentition period. An extraoral examination did not reveal any serious facial asymmetry. An intraoral examination revealed the presence of his deciduous upper right canine in the arch, the absence of the corresponding canine, and microdontia of his upper lateral incisors. His left lateral incisor showed a crossbite relationship with his lower left canine. A class I molar interocclusal relationship was present on his right and left sides, with minimal crowding in the front section of his lower arch (Figure 1). No notable events were evident from our patient’s or his family’s medical history that could be correlated with the altered tooth eruption or position.

**Figure 2:**
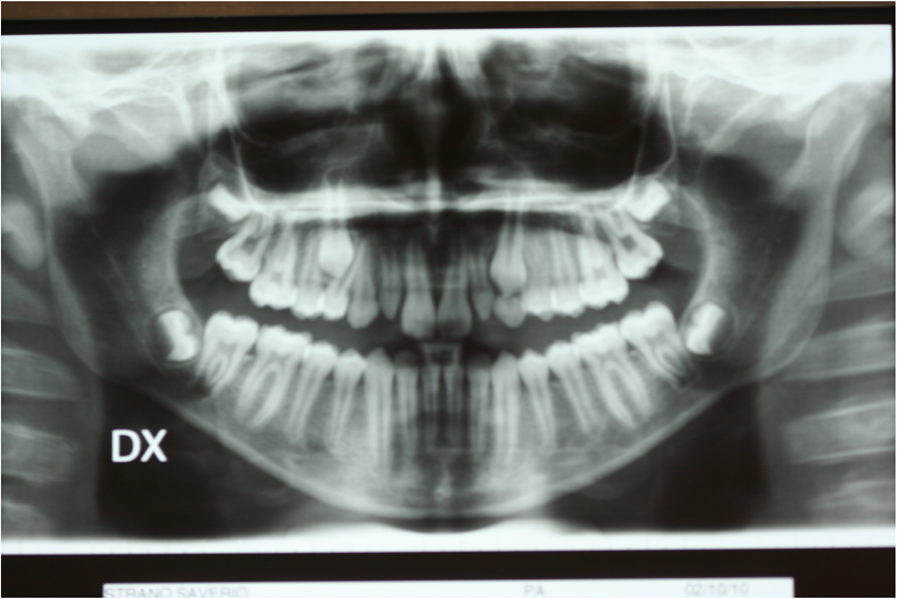
**Pretreatment orthopantomography.**

To determine an adequate treatment plan, our patient underwent orthopantomography of his arches and latero-lateral cranium teleradiography for cephalometric evaluation. The orthopantomography highlighted the retention of his right upper canine and its transposition with his first premolar (Figure 2).

One possible treatment approach for this patient was to align his teeth into the transposed position. Although this approach would probably have required less time overall, it had some disadvantages in terms of aesthetics and occlusion. Therefore, a combined surgical-orthodontic treatment was selected, with the aim of correcting the transposition and aligning the teeth into their correct positions. The selected approach involved a surgical incision in the mucosa proximal to the retained and transposed canine, traction of the tooth in the dental arch into its physiological position using an anchorage device, and banding of the dental arches to obtain alignment and leveling. The proposed treatment was interceptive and was chosen to prevent further impaction of the canine into the first molar.

**Figure 3:**
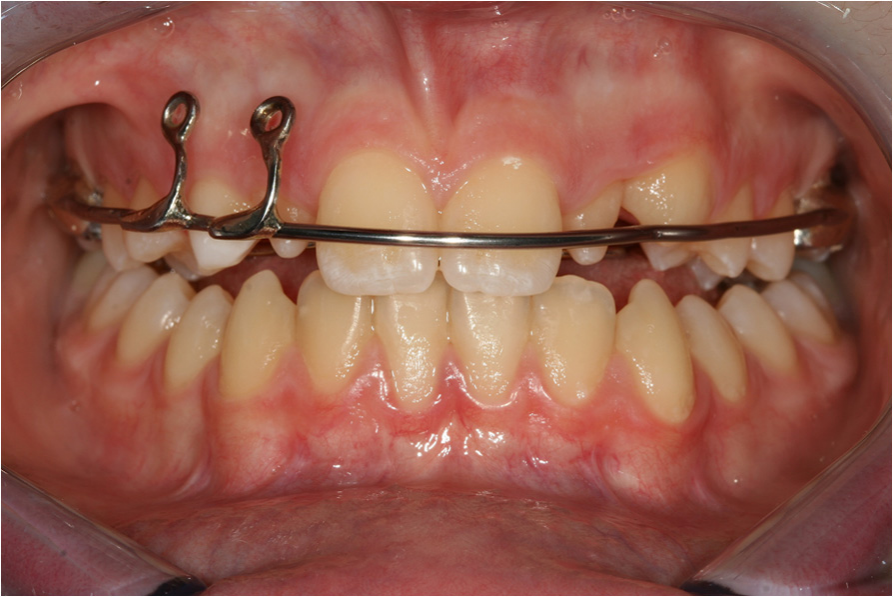
**Surgical operation.**

**Figure 4:**
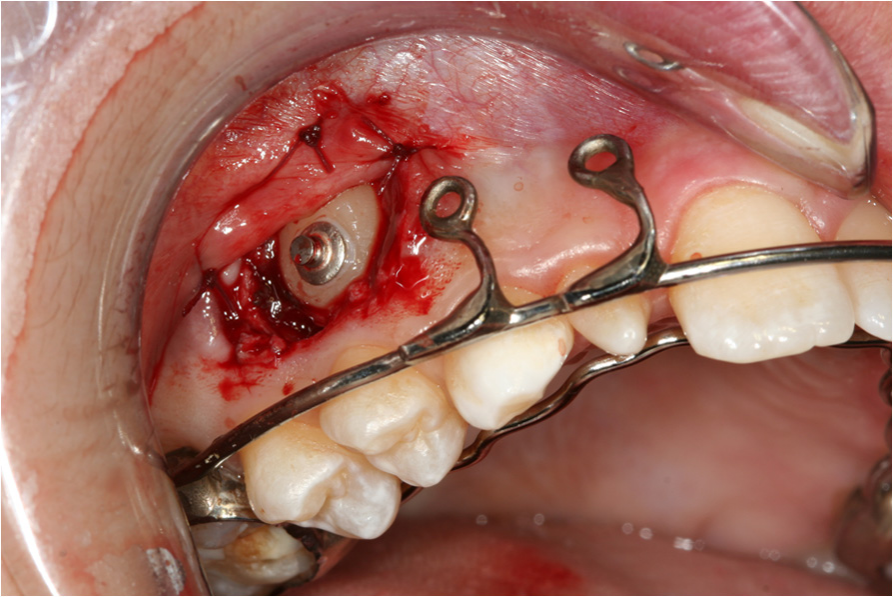
**Splint with eyelet positioned and cemented.**

During the first session, a dental technician used a band to take an impression of our patient’s upper teeth, creating a splint with two eyelets in zones 12 and 13 to ensure traction of the canine in the arch. The splint was cemented (Figure 3) and, one week later, surgery was performed. The oral surgeon made an incision in the mucosa to expose the crown and created a trapezoidal paramarginal flap (Figure 4). A button was placed at the crown level and tied with an elastic wire to the more distal eyelet of the splint to start the traction. The deciduous canine was preserved to maintain the necessary space for repositioning the permanent tooth. The elastic wire was replaced approximately every 15 days to ensure a slow and constant traction, in such a way as to avoid damage to the periodontal tissue and the canine.

**Figure 5:**
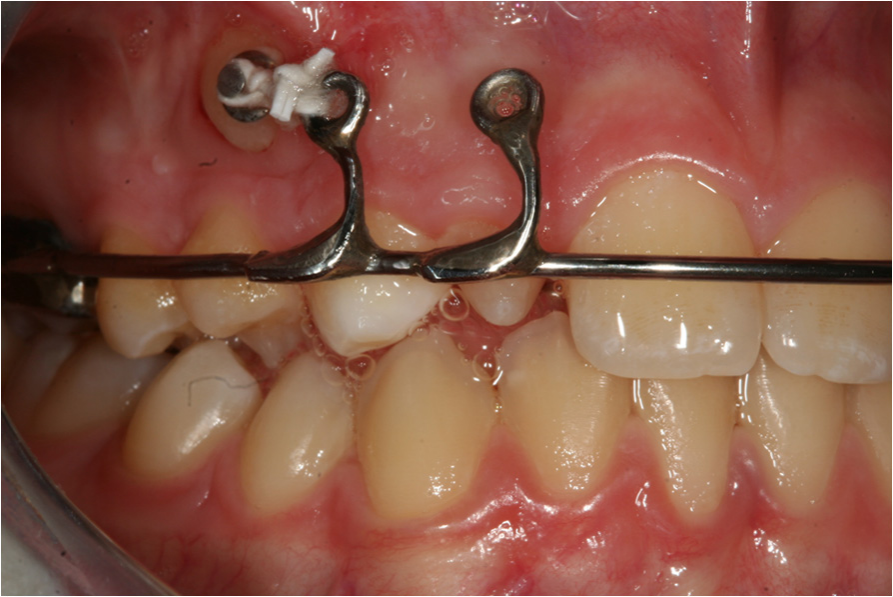
**Intraoral photo two months after the start of traction.**

**Figure 6:**
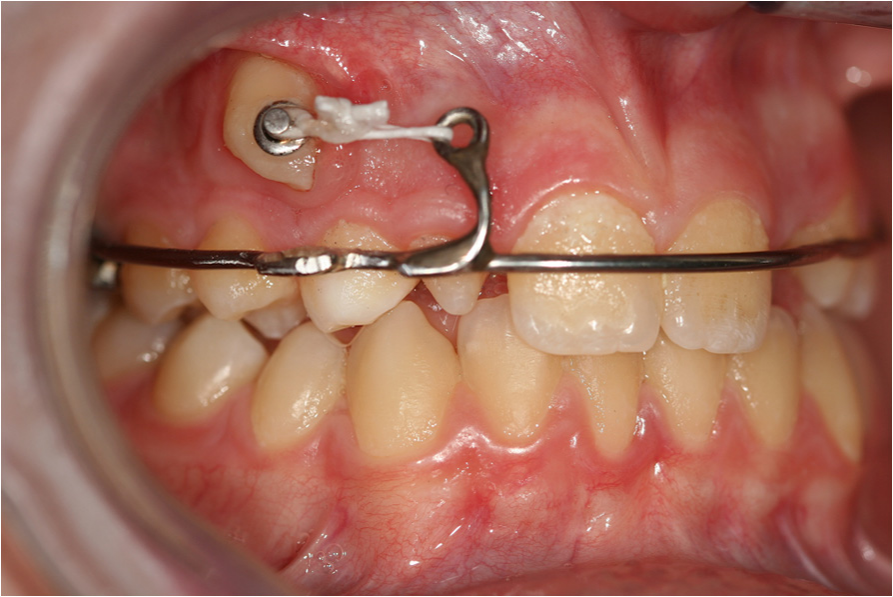
**Intraoral photo four months after the start of traction.**

About two months after surgery, his tooth was visible in the arch (Figure 5). Traction was continued by tying an elastic wire to the mesial eyelet of the splint in zone 13. The more distal eyelet was removed. Four months after surgery, the tooth was sufficiently visible (Figure 6) to allow for removal of the splint, extraction of the deciduous canine, replacement of the button with an orthodontic brace, and banding of the arches.

**Figure 7:**
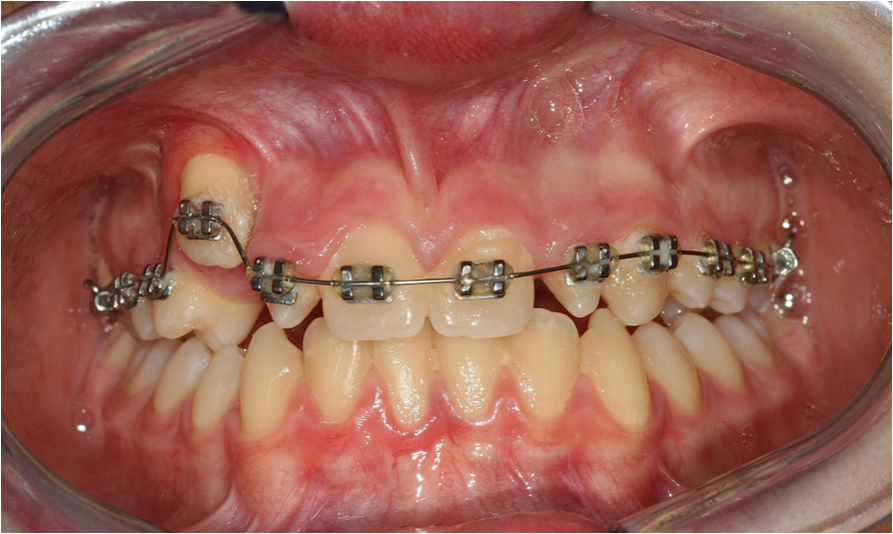
**Banding and bonding of upper arch.**

Bonding was performed with pre-torqued and pre-angled brackets with a 0.022-inch slot. The first arch used was a 0.014-inch nickel-titanium round arch, to which the canine was directly tied (Figure 7). About two months after the start of treatment, a 0.018-inch nickel-titanium arch was applied. Once the crowding was resolved, the intermediate stage was begun, and a 0.016×0.022-inch nickel-titanium arch was applied. For the final stage, a 0.019×0.025-inch steel arch was used.

**Figure 8:**
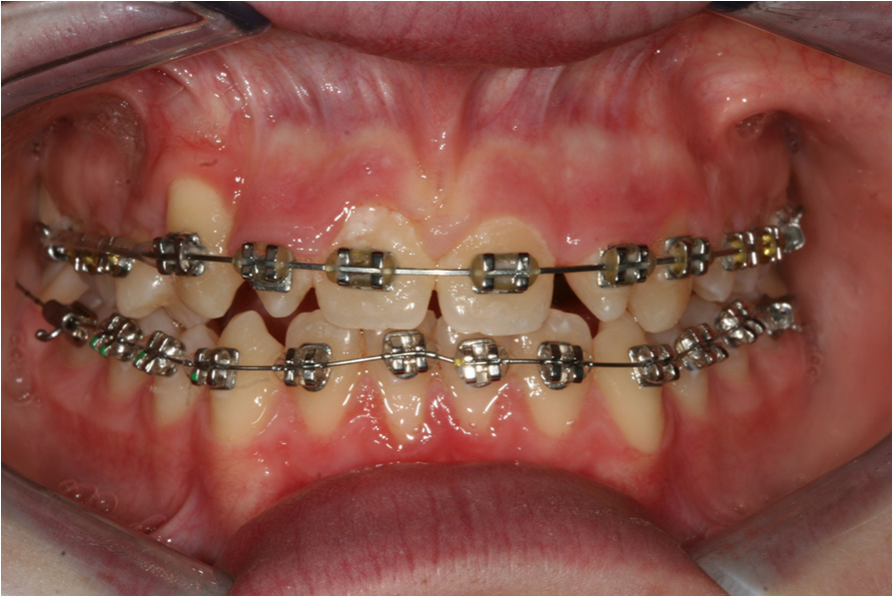
**Banding and bonding of lower arch.**

**Figure 9:**
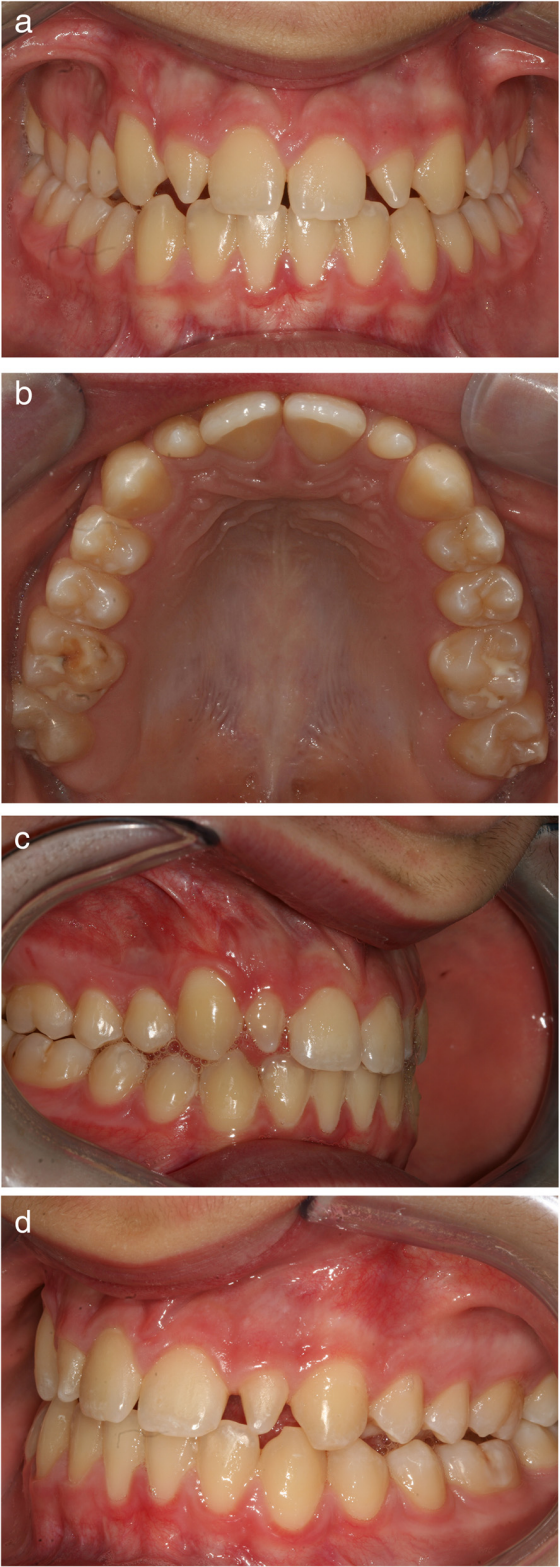
**Post treatment intraoral photograph. (a)** Frontal view, **(b)** maxillary occlusal view, **(c)** right side, **(d )** left side.

As soon as the canine reached its correct position in the arch, the lower arch was banded. The same procedure used for the upper arch was applied, using the same braces and the same sequence of wires (Figure 8). Banding was removed after the established objectives were met, that is, the transposition was corrected and his arches were aligned and leveled (Figure 9).

Our patient’s treatment lasted for two years. At the end of the treatment, he had a good aesthetic outcome. The median lines of occlusion were centered, his molars were in a class I relationship, and his right upper canine showed a slight gingival recession. The microdontic lateral incisors will be aesthetically reconstructed at a later stage, and a decision will be made then as to whether the reconstruction will be direct or indirect.

## Conclusions

Transposition is an anomaly of tooth position, the most frequent of which involves the canine and the first maxillary premolar. The most suitable treatment depends on the occlusion, level of dental crowding, aesthetics, position of the radicular apices, and specific needs of the patient. In this case, we opted for definitive treatment with orthodontic alignment of the transposed teeth into their physiological position. The treatment allowed us to recover the permanent canine from the transposed position and reposition it into its natural position. Our patient is satisfied with the aesthetic results obtained.

## Consent

Written informed consent was obtained from the patient’s guardian for publication of this case report and any accompanying images. A copy of the written consent is available for review by the Editor-in-Chief of this journal.
