# Correction to: Orthodontic treatment of the transposition of a maxillary canine and a first premolar: a case report

**DOI:** 10.1186/s13256-019-2016-9

**Published:** 2019-03-04

**Authors:** Maria Teresa Dinoi, Stefano Mummolo, Annalisa Monaco, Enrico Marchetti, Vincenzo Campanella, Giuseppe Marzo

**Affiliations:** 1 Department MeSVA, School of Dentistry, University of L’Aquila, L’Aquila, Italy; 2 Department of Clinical Science and Translational Medicine, University of Rome “Tor Vergata”, Rome, Italy

**Correction: J Med Case Reports (2015) 9: 48**

**https://doi.org/10.1186/s13256-015-0521-z**

In the publication of this article [1], there is an error in the Family Name and Given Name of the authors since these were interchanged.

The error:

Dinoi Maria Teresa^1*^, Mummolo Stefano^1^, Monaco Annalisa^1^, Marchetti Enrico^1^, Campanella Vincenzo^2^ and Marzo Giuseppe^1^

Should instead read:

Maria Teresa Dinoi^1*^, Stefano Mummolo^1^, Annalisa Monaco^1^, Enrico Marchetti^1^, Vincenzo Campanella^2^ and Giuseppe Marzo^1^

This has now been included in this correction.
